# Connect perspective of mixed methods research and systematic observation of participatory interaction

**DOI:** 10.3389/fpsyg.2026.1937320

**Published:** 2026-10-22

**Authors:** Conrad Izquierdo, Héctor Tronchoni

**Affiliations:** 1 Faculty of Psychology, Universitat Autònoma de Barcelona, Barcelona, Spain; 2 Faculty of Philosophy and Education Sciences, Universitat de València, València, Spain

**Keywords:** integration, interaction analysis, interpersonal communication, mixed methods, sequential data, systematic observation

## Abstract

The aim of this article is to address the mixed methods dimension of systematic observation in the study of human communication, placing the focus on the *quantitizing* of qualitative data. In the introduction we highlight the contribution of *M. Teresa* Anguera as the promoter and director of the structured proposal of observational methodology committed to objectivity, rigor, transparency, and the generalizability of systematic studies of behavior. The second section condenses a few central elements of the ongoing complex dialogue in the debate about the “qual-quan” integration problem in mixed methods studies. The capture of this dialogue moment focuses on the active and productive positions which show a critical interest in the mixed methods approach of observational methodology that combines qualitative and quantitative elements in one single method. The third section deals with the adaptability of the third paradigm and of the systematic observation studies within the disciplinary field of interpersonal communication. In the fourth section, the content underpins six vertebral steps of the methodological decisions linked to the structuring process of systematic observation. The theoretical-methodological content and its contributions belong to four descriptive-evaluative doctoral dissertations about participative dialogic behavior within the context ordinary tasks. Finally, in the discussion, we highlight the efforts made in training researchers committed to the development of observational methodology in accordance with the updated agenda of its challenges. One of these is to promote its presence with optimized contributions useful for the understanding and improvement of dialogic competence in daily life.

## 1 Introduction

The call to systematize the observational studies of behavior in natural situations (Weick, 1968) was heeded in our country by *M. Teresa* Anguera in the 1970s (Anguera, 1979). Her initial impetus evolved into the setting up of an inter-university research group, Research Group and Innovation in Designs (GRID, https://webs.uab.cat/grid), which has since received the growing and active support from a network of both experienced and trainee researchers in different countries and universities all over the world.

In terms of the reputation of the scientific quality of structured observational methodology in all its phases, this was achieved via a widely disseminated bibliography of projects, both completed and in progress. This has materialized in the special recognition dedicated to *M. Teresa* Anguera by the scientific community to which she belonged, and by other prestigious institutions and social entities (see Figure 1).

**Figure 1:**
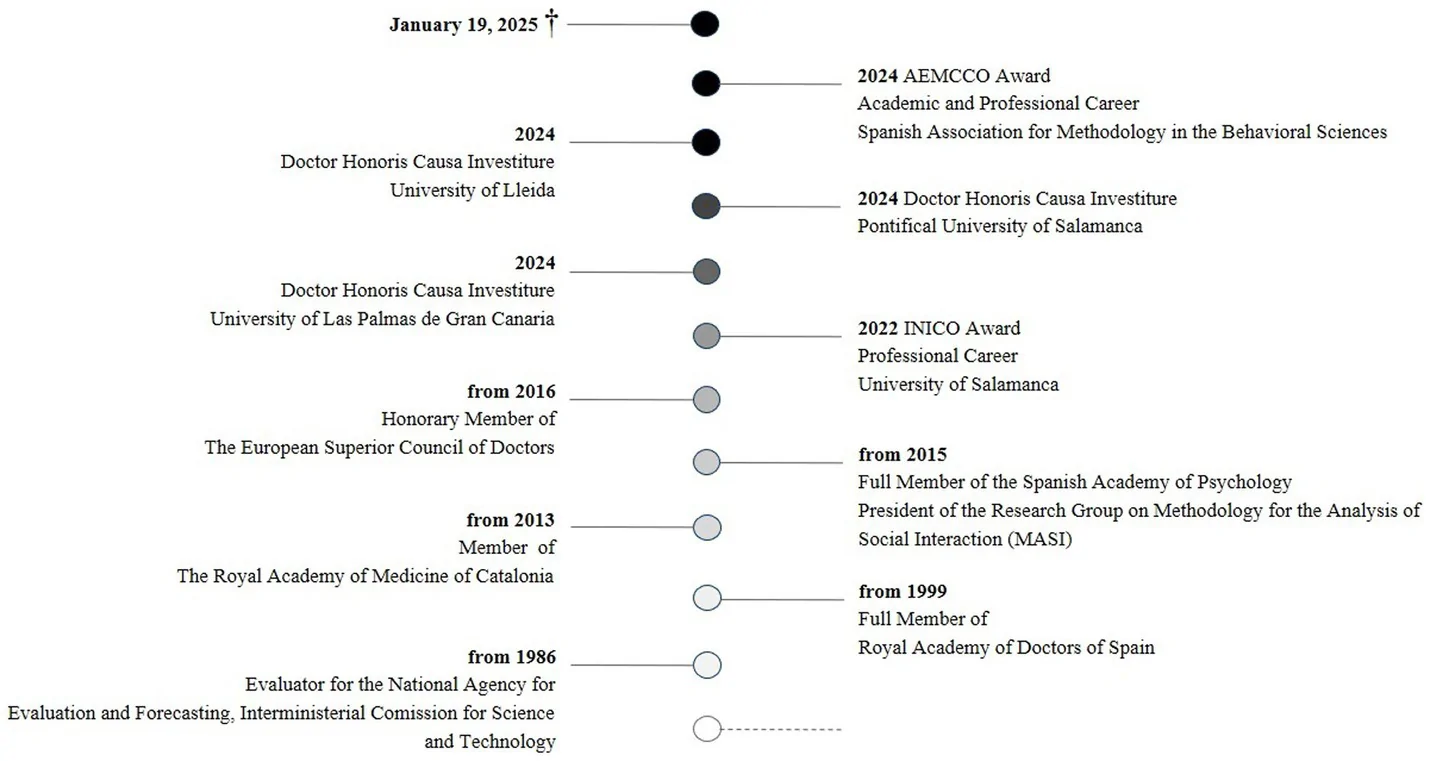
Excerpt of acknowledgments of the scientific work of *M. Teresa* Anguera. Compiled by the authors based on https://raed.academy/academicos/dra-maria-teresa-anguera-argilaga/

Therefore, and in response to the exhortation from Johnson and Onwuegbuzie (2004, p. 16), the aim of this article is to share the application of the systematic observation method, identified as a mixed methods approach, within the disciplinary domain of interpersonal communication and of group interaction analysis methods.

“It is time that methodologists catch up with practicing researchers! It is now time that all researchers and research methodologists formally recognize the third research paradigm and begin systematically writing about it and using it” (Johnson and Onwuegbuzie, 2004, p. 16).

As we have outlined in the abstract, the main body of this article consists of three sections that trace a route going from systematic observation as a scientific methodology with a mixed methods approach, to the summarized presentation of the common denominator of structured methodological decisions from four published doctoral theses about the systematic observation of participatory interaction. The intermediary step could be conceptualized as the necessary critical recognition of mixed methods studies with observational follow-up design, within the disciplinary domain of reference.

## 2 Mixed methods integration in observational methodology: connecting two data sets

Observational methodology has addressed the basic questions that form part of the ongoing complex debate within the Mixed Methods International Research Association (MMIRA, https://mmira.wildapricot.org/) community (Mertens et al., 2016) concerning the central problem of integration (Anguera, 2024; Anguera et al., 2018a, 2020, 2021a, 2023). The reliable integration of quantitative and qualitative elements at various levels is the dimension that legitimizes the concept of the mixed methods approach (Fetters et al., 2013; Schoonenboom and Johnson, 2021).

Bazeley (2009, 2018) asserts that it is both beneficial and essential to use mixed methods strategies for data collection and analysis when studying the experience and behavior of people, both as individuals or in a group. This conviction, promoted by the founders and inspirers of the MMIRA community, perpetuates the need to go deeper into the methodological integration of qualitative and quantitative elements (Bryman, 2006).

As a result, there is an ongoing expanding task to formulate criteria for assessing the integrating coherence of the mixed methods design applied in each empirical study linked to the third paradigm. Our reference point is Bazeley’s critical assessment of the mode of integration of qualitative and quantitative elements in the observational designs that favor a diachronic analysis of qualitative data (Anguera et al., 2021b).

Bazeley (2018) validates systematic observation as a mixed methods strategy and places the quantitative analysis of observational data within the group of hybrid strategies, a term taken from the automotive sector. The implicated processes are the structured record of the coding of behavior perceived in natural contexts over time and its transformation for statistical or mathematical analysis. In the conceptual model of integration dimensions that Bazeley (2024) proposes, data transformation aligns with the *connect* strategy proposed by Creswell and Plano Clark (2011, p. 7): “connecting the two datasets by having one build on the other”.

Integration, understood as the transactional relationship between the qualitative and quantitative components of a study, challenges the “qual *vs.* quan” dichotomy when it comes to addressing the factual content (physical reality and inter-subjective reality) of the research questions (Izquierdo and Anguera, 2021).

Onwuegbuzie et al. (2018), in a basic approach based on Bayesian statistics, use the notation 1 + 1 = 1 to suggest that complete integration is committed to a wide variety of factors that interact reciprocally in data collection, analysis, and interpretation. For example: the context, the findings of previous research, the historical trajectory, the sample, etc.

Thus, leaving out the pressure of the “qual *vs*. quan” dichotomy leads researchers to believe in the importance of having different perspectives, which is much richer given that different data collection and analysis procedures can be used, enabling a complete integration (Creamer, 2018). However, we must be alert to the difficulties which could be argued when addressing the different points of integration and of addition in a mixed methods study (Schoonenboom, 2023).

It is beyond the limits of this article to go into the controversy of data transformation seriously, without searching for the easy path of simplification. Observational methodology in this subject matter retains its demanding position, in the sense of continuing to explore the dialogue space between the two great paradigms about the nature of interdependence in the “qual-quan” continuum (Anguera, 2024).

On the other hand, we are aware that we work in an environment where there is basic agreement among dissenters who support the need to bring together committed systematic forces with a pragmatic vision of method integration (Johnson and Onwuegbuzie, 2004). Hence we can sidestep the debate and go straight on to conceptualize the “QUAL-QUAN” transformation process of systematic observation (Anguera et al., 2020; Portell et al., 2015a).

The scientific procedure that endorses the connect integration modality in an observational study corresponds to the “QUAL-QUAN” transformation of the “QUAL-QUAN-QUAL” chain. The “QUAL-QUAN” phase, known as *quantitizing,* centers on the order and duration parameters of behavioral events (Bakeman, 1978). Figure 2 shows the set of aspects to take into consideration for verification of the “QUAL-QUAN-QUAL” methodological procedure.

**Figure 2:**
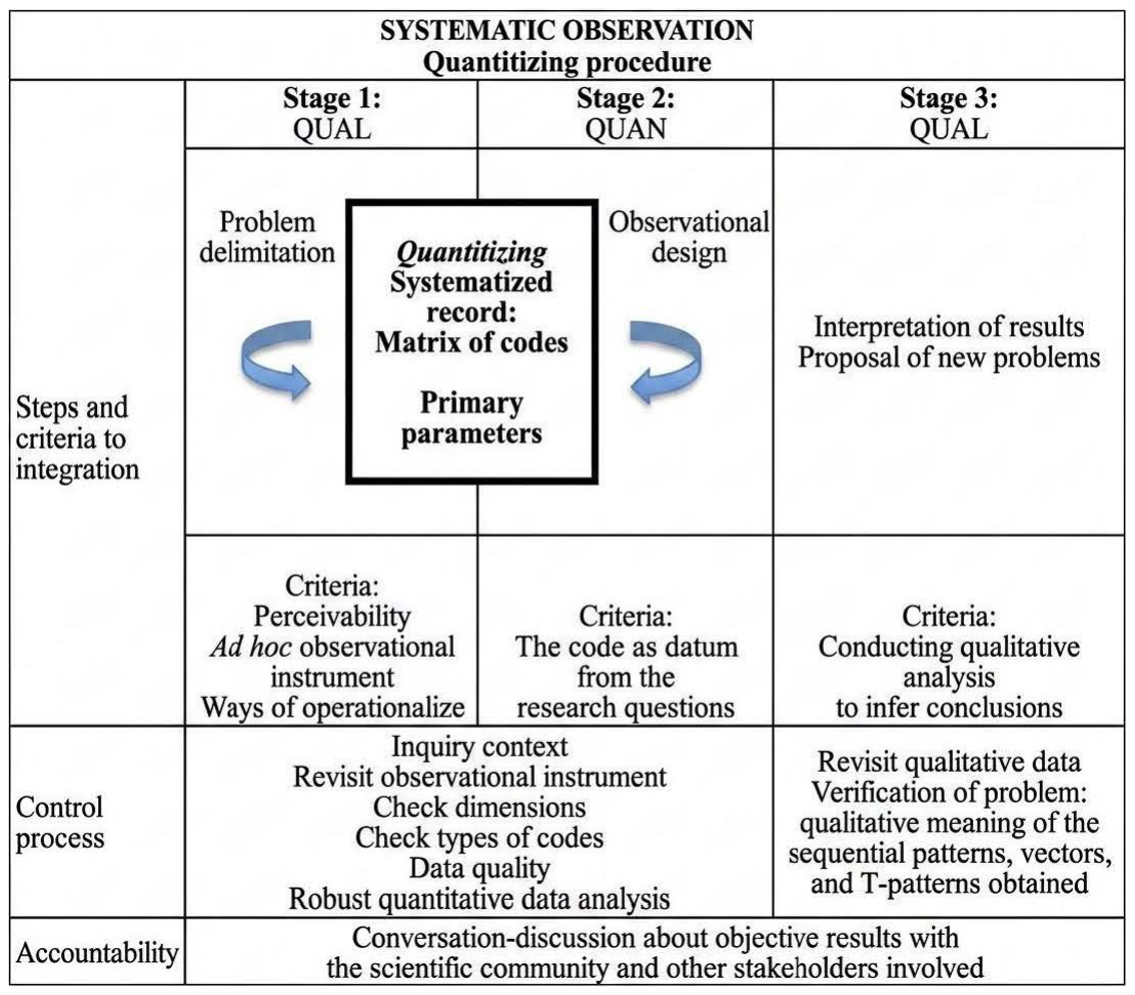
The QUAL-QUAN integration process. Source: Izquierdo and Anguera (2021).

## 3 Systematic observation and interpersonal communication

The scientific approach of the study of communicative behavior has been characterized by being, in general terms, a systematic, controlled, empirical, and critical inquiry into the presumed relationships between natural phenomena. On the other hand, the innovative approach of ethologist Patrick Bateson (Bateson and Martin, 2021) to the problem of a behavioral measurement has provided a guide which sustains and inspires the integration of qualitative and quantitative elements on different levels: the aim of the study, the research questions, and the analysis plan.

Bearing these references in mind, we will go on to place the mixed methods approach of systematic observation within the disciplinary domain of interpersonal communication and the analysis of group interaction.

### 3.1 Our disciplinary research setting

Linking communication to social participation has implied a shift in the interpersonal conception of human communication provided by the incorporation of epistemological anthropology problems via the work of Bateson (1972).

The theoretical and empirical study of communication is treated as a process of bidirectional, transactional, and regulating interaction, made up of multi-sign units (verbal and non-verbal) and with a multilevel organization of deliberate and expressive messages in the cultural context within which the actors in the communicative encounter operate.

This ecological network vision of communication and change through interaction (2 + n individuals in their environment) has evolved, as Gregory Bateson points out (Ruesch and Bateson, 1951), towards studying a communicative encounter as:

- An open interactive system,
- Centered on diachronic and process statements,
- With a combined interdisciplinary approach on epistemological and methodological levels,
- Placing emphasis on the rules that allow us to represent knowledge, on the identification of patterns, and on the graphic representations that shape causal and circular relationships.

In effect, the pragmatic attitude or acceptance of the terms of scientific realism by researchers of interpersonal communication has been substantiated by placing value on the interdisciplinary nature and equality of the different methodologies, since there is no single privileged approach for the production and use of scientific knowledge (Izquierdo and Anguera, 2021).

The shared declarations in this sense have contributed to: (i) showing the usefulness and value of both methodologies, and (ii) accepting a reasonable doubt about the veracity of the controversy between research methodologies.

Nevertheless, Levine (2011) maintains that *“…*doing both qualitative and quantitative research may well be too ambitious for many mere mortals*”* (p. 28). We understand that the “ambition” is reasonable when researchers have access to training and supervision mediums which guide the systematic observation process step by step, as in our case, and develop an awareness of the reach (strengths and weaknesses) of the methodological decisions that structure the object-method relationship (see Discussion).

### 3.2 Our studies of participatory interaction

Based within the environment of doctoral research focused on the systematic observation linked to the analysis of interaction (Brauner et al., 2018), our interest focuses on the following communicative situations: the review conversations of cohabiting couples (Cuervo, 2014), organizational meetings of professional teams (Izquierdo, 1993/1995), survivor therapy support groups (Roustan, 2010), and the university expository lecture (Tronchoni, 2019). In all these studies, conversational dynamism, the construction/transformation of the relationship link, and the accumulative character of the exchanges are present in the demand for personal information, in the search for agreements, in the assisted taking of individual decisions about how to channel the new future, or in the enabling of significant learning.

Two key factors justify the selection made from the dissertations listed in Table 1. First, the co-authors of this paper wrote two of these dissertations, and one of us co-supervised the remaining three. On the other hand, the four studies share the key concept of management of the attentional space generated by the use of the spoken word in conversations. This attentional space guides and constrains the interactive behavior within the dialogic task context (Izquierdo, 1993/1995): When to speak?, for how long?, how frequently?, do I interrupt?, etc.

**Table 1:** A list of dissertations applying the quantitative analysis of communicative interaction to diachronic qualitative data.

Num.	Authors	Year of defense	Title	Supervisors
1	Vicenç Quera Jordana	1986	Mother-Infant Interaction in Non-Human Primates	M. T. Anguera Dr. Jordi Sabater
2	M. Luísa Herrero Nivela	1989	Impact of Personal History on Classroom Behavior: An Analytical Observational Study	M. T. Anguera
3	Lluís Farré Grau	1990	Emotional Dynamics in Therapeutic Care for Neoplastic Patients: Analysis of Anxiety and Defensive Resources Through Direct and Indirect Systematic Observation	M. T. Anguera Dr. Víctor Hernández
4	Mercè Mitjavila García	1990	Observational Study of Mother-Infant Interaction in the First Two Months of Life	M. T. Anguera Dr. Edelmira Doménech
5	Elisa Cucala Campos	1991	Idiographic Structural Configurations Determinant of Non-Verbal Communication in Non-Attentional Parameters in School Settings	M. T. Anguera
6	Marta Castañer i Balcells	1992	Non-Verbal Communication of the Physical Educator: Construction of a Categorial System for the Observation and Analysis of Kinesic Behavior	M. T. Anguera
7	Palmira Pérez Vázquez	1992	Analysis of Interactive Behavior in School Settings Using Flanders’ System: Historical Evolution, Application, and Dissemination Within Different Observational Systems	M. T. Anguera
8	Juan Carlos Tójar Hurtado	1992	Concordance of Observational Records in Sequential Data: Applied Research in Classroom Settings	M. T. Anguera
9	Conrad Izquierdo Rodríguez	1993	Assessment of Teachers’ Communicative Skills in the Educational Context of a Primary School Experimental Center: A Study of Turn-Taking Systems	M. T. Anguera
10	Oleguer Camerino Foguet	1994	Educational Interaction in Physical-Recreational Activities for the Elderly: A Methodological Integration Analysis	M. T. Anguera Dr. Jaume Trilla
11	Antonio Hernández Mendo	1996	Observation and Analysis of Game Patterns in Sociomotor Sports	M. T. Anguera Dr. Ramón Arce
12	Gisela Riberas Bargalló	1998	Observational Study of Educator-Infant Communicative Interaction in a Reception Center	M. T. Anguera Dr. Mercè Mitjavila
13	Eulàlia Arias Pujol	2004	Observational Study of Interaction in a Psychotherapy Group Based on Conversation Analysis	M. T. Anguera Dr. Maite Miró Coll Dr. Eulàlia Torras Armangué
14	Marina Roustan	2010	Observational Study of Interactive Help in Breast Cancer Post-Treatment: GAFA Meetings	M. T. Anguera Dr. Conrad Izquierdo
15	Juan José Cuervo	2014	Communication and Sustainability in Viable Couples: A Multiple Case Observational Study	M. T. Anguera Dr. Conrado Izquierdo Dr. Juan Luis Linares
16	Alejandra Barbado Sánchez	2016	The Efficacy of Animal-Assisted Therapy in People with Disabilities: Analysis of Communicative Flows Between Individuals with Autism Spectrum Disorder and Therapy Dogs	M. T. Anguera Dr. Miguel Ángel Verdugo Alonso
17	Héctor Tronchoni Albert	2019	Observational Study of Multimodal Communication in the University Classroom: Contexts and Structures of Discursive Participation in Lectures	M. T. Anguera Dr. Conrad Izquierdo
18	Ivette González Sarkis	2020	Impact of Prematurity on Communicative Interaction in the Mother-Infant Dyad and on Maternal Self-Perception of Stress	M. T. Anguera Dr. Mercé Mitjavila
19	Luca Del Giacco	2021	The Mutual Regulation Processes During the Psychodynamic Psychotherapy of Depression: An Observational Study of Communicative Acts in the Construction of the Early Therapeutic Alliance	M. T. Anguera Dr. Silvia Salcuni
20	Marina Alarcón Espinoza	2021	Emotional Self-Regulation in Everyday School Life: Systematic Observation in Classrooms with Chilean Students Aged 10 to 12	M. T. Anguera Dr. Paula Samper
21	Álvaro Moya	2024	TEDPatt: Communicative Behavior Patterns in Top TED Speakers. Observational Methodology Applied to Innovative Scientific Outreach Communication Processes	M. T. Anguera Dr. Elena Herrán

Compiled by the authors based on https://raed.academy/academicos/dra-maria-teresa-anguera-argilaga/ the original titles have been translated into English.

Consequently, the conversational activity carried out by the participants in these examples demands that the flow of exchanges is done mainly in the structured transactional interactive mode, such as *turn* and *change of turn* (Holler et al., 2015). The golden rule is: we have to listen with a view to *achieving* the aim of the encounter (Floyd, 2010). The attentional space generated by the use of the spoken word provides information on the characteristics of dialogic interaction.

In terms of the temporary setting dictated by the research questions, our general queries were: (i) how do the participants drive the dialogic dynamic of the oral task throughout the duration of the encounter? and (ii) what information and/or relationship do they share from one moment to the next?

The evaluative aim of these two broad questions covers: (i) the problems of influence in the way cooperation occurs in the fluid organization of dialogic interaction, and (ii) the problems of mutual adaptation in the way collaboration takes place in maintaining the commitment to the task of the encounter.

It follows that the intervention which could result from the scientific report (Portell et al., 2015b) would be conceived in terms of: improving the performance of interpersonal communicative competences, identifying and becoming aware of the complexity of the abilities under consideration, and/or proposing the creation and follow-up of strategic plans to be developed in accordance with the nature and reach of the demand of the intervention. In Table 2 we show the selection criteria of the dissertations included in Section 4 of this article.

**Table 2:** Selection criteria of the dissertations.

Criteria	Dissertations (see Table 1)			
Issue	Review durability of their marital life (Cuervo, 2014)	Teachers’ coordination meeting (Izquierdo, 1993/1995)	Interactive support group with women survivors (Roustan, 2010)	Lectures in university classrooms (Tronchoni, 2019)
Social relevance	Long-term couples: characteristics of their dialogic activity	Understanding constructive participation in joint decision-making	Social aid group with women who have undergone breast cancer	Improve to lecture format
Pertinence of the study	Interaction analysis: Marital conversation task	Interaction analysis: Group discussion	Interaction analysis: Group therapy	Interaction analysis: Discursive and semiotic learning strategies
Methodological structuring: Systematic Observation (see Section 4)	Design I/S/M *Ad hoc* observation instrument Quality control Analysis of temporal regularities	Design N-I/S/M Ad hoc observation instrument Quality control Analysis of temporal regularities	Design I/S/M Ad hoc observation instrument. Quality control Analysis of temporal regularities	Design N-I/S/M Ad hoc observation instrument. Quality control Analysis of temporal regularities
Theoretical-methodological contribution	Comprehensive model of communication and the durability of the marital relationship	Ideal meeting model observed as an extended turn system	A GAFA (analytic, formative, and social aid) therapeutic matrix	Participatory listening matrix of interactive learning

## 4 From the complexity of the perceived object to data analysis

The methodological complexity to consider in a single study with only one method is the symmetric integration of qualitative and quantitative elements. We have dealt with this issue following the logic of the observational methodology process that allows us to sequence both perspectives forming the QUAL-QUAN-QUAL chain.

### 4.1 Constellation of key decisions

*Perceivability of the object*. Our conceptualization of the non-intrusive systematic observation method in the current of interactive behavior places the scientific observer in the position of transducer (Barker, 1968). That is, their work consists of reliably transcribing the observed socio-behavioral phenomenon into the categorized language of the scientific field of reference, and analyzing the obtained data within the framework of the adopted theory.

We assume that the perception of a “I-you-here-now” goes beyond the positive transcription of the (co)presence relationship of two or more people subordinated to a specific context (Vion, 1996). The conjugated orientation that identifies the encounters between participants who look at and hear (and much more) each other is an indicator of the implication that distinguishes committed oral interaction from disperse interaction (Goffman, 1964).

In fact, the participants’ relationship marked by the dialogue is fed by the inter-play between the pre-established rules and the constructive action (of the “do” dimension) of the oral exchanges carried out. In short, the oral dialogic task that identifies each encounter is fueled (or not) by relational actions, and advances (or not) with instrumental actions on an informational level (Bales, 1950).

Given that types of dialogic encounters between people are very diverse, we have focused on speaking and listening activities, performed alternately and simultaneously, with a clear shared informative aim that is not to be confused with gossip, post mortem or ceremonial conversations, or other kinds of small talk.

Therefore, the formality of taking turns and changing turn when speaking – a mechanism that guarantees dialogic alternation (Duncan and Fiske, 1977) –, is used more or less freely by the participants in the encounters or is marked by the interlocutory situation: the speakers’ inner moment, rules and constricted idiosyncratic obligations, and the institutional code of reference (Duncan and Fiske, 1985; Poyatos, 1983).

The temporary qualities of the turns system, that form part of the set of temporary qualities specific to the process of interpersonal communication (Werner and Haggard, 1985), on the one hand widens the focus of the participatory interaction by highlighting what each participant can do for the other speakers in behaviorally defining the act of their contribution in terms of order, duration and rhythm. Speech rhythm takes into account speed, pauses, silences and tone of voice.

On the other hand, the structuring of the speaking turn includes the temporary characteristics of the covered topics, along with the temporary restrictions that depend on the conversational situation.

*Observational design.* In accordance with the criteria of the observational designs used in the aforementioned studies of participatory interaction (Anguera et al., 2001), we observed the dynamics (intra-sessional and inter-sessional follow-up) of the transactions (multidimensional configurations). Dyads, triads, and small or medium groups were considered as idiographic units, whether the participants were members of a defined social structure (couple, family, professional team, therapy group, classroom), or nomothetic units (number of participants > 1). Both options – description of the participants’ behavior and description of the group – are compatible in a single study, albeit separately. The normalized notation of this design is: I-N/S/M (Idiographic-Nomothetic, S = Follow-up, Multidimensional).

*Segmentation of the continuum.* Behavior consists of a continuous flow of movements and events. We assume that the shortest identifiable transaction in the viewing of the session recording is a who-to-whom turn lasting for 1 s, and that each second of recording can be split into FPS units if the necessary technology is available. The presence of voice was considered as a structural indicator that the conversation was activated and not finished (are they talking?).

*Ad hoc observational instruments: syntax and identifying categories*. Why *ad hoc*? Note that the idea is not to suspend the general rule of finding a coding scheme that adapts to the structuring of the research object, but rather to understand the need to build a systematic observation instrument in itself (Tschan et al., 2018).

The extended coding of structures and communication in the dialogic interaction that incorporates the qualitative and quantitative consequences of turn/change-of-turn behavior comprises the following (Poyatos, 1983): external environmental components and behavior based environmental components, along with internal components of the multimodal transactional interaction (linguistic, paralinguistic, and kinesthetic) in the real situation of the considered conversational encounters. Counting on the presence of overlapped speech and multiple turns, the identifiable defined turns – who speaks/listens to who listens/speaks – describe three dialogic positions: *holds the floor*. *Auditor back-channel and supporting speaker* (Duncan and Fiske, 1977; Knapp, 1980; Poyatos, 1983; Rosenfeld, 1978).

The temporary order imposed by the intra-sessional follow-up of the dialogic sequence is incorporated into the observation instrument using the field format structure. The diachronic reading of the succession of configurations is combined with the synchronous reading of the hierarchical dimensional criteria of the observation. In our case, the adopted observational design includes the multidimensional sequential description of the synchronous configurations moment by moment; i.e., the data we are seeking in order to answer to the proposed research questions imply different coding levels for each criterion dimension considered and itemized into sub-dimensions. The conceptual and interpretative component lies in the primary measurements and in the category systems defined as Exhaustive and Mutually Exclusive systems (E/ME). Table 3 outlines the systematic observation process: QUAL-QUAN phase (I).

**Table 3:** Systematic observation process: QUAL-QUAN phase (I).

Systematic observation process: QUAL-QUAN phase (I) Common and differential elements			
Issues			
Durability review of their marital fife (Cuervo, 2014)	Teachers’ co-ordination meeting (Izquierdo, 1993/1995)	Interactive support group with women survivors (Roustan, 2010)	Lectures in university classrooms (Tronchoni, 2019)
Perceivability of the object			
Dialogic description of the conversational task, perceived as alternating turn-taking transactional units (unmarked and/or marked) in which the fluid and committed management of exchanges is negotiated at both the content and relational levels.			
Observational design			
I/S/M	I-N/S/M	I/S/M	I-N/S/M
Ad hoc observational instruments: *Field format dimensions #Number of basic level categories E/ME			
*Primary qualities of the turn *Uses turn-taking *Feedback on maintaining contact *Relational warmth #17	*External events *Primary qualities of the turn *Turn-taking process *Maintaining contact process #15	*Primary qualities of the turn *Structure turn-taking *Relational process-support task *Therapist’s intervention #26	*Primary qualities of the turn *Organizing contributions of interaction *Participatory regulation #45
Operationalization			
Alternation dimension: Order (example). It provides measurable information on the frequency of a coupling, the sequential recording structure, and the positioning of the contribution within the discursive structure of the dialogic task’s communicative format. Depending on the setting in which the observation takes place (couple conversation, meetings, therapeutic groups, classroom groups, etc.), other aspects of the order of alternation to be measured are also selected. For example: establishing a silence/passivity index in lecture-based classes, or a group awareness index in coordination meetings.			
Control in the construction of the category systems			
Obtaining a preliminary list of descriptive terms subjected to conceptual analysis. (A combination of an essential theoretical review on the topic and passive observation of the phenomenon under study is recommended). Selecting the categorical terms that contribute most to providing a precise, consistent, and verifiable answer to the research questions. Verifying at multiple stages of the instrument construction process that each selected category complies with the essential attributes of the conceptual core. (Behaviors assigned to the same refined category may exhibit a degree of heterogeneity in their way of expression). Verifying that unidimensional E/ME categories have been attained for each criterion dimension of the field format.			

*Data analysis.* The multi-event turn-taking transactions that are observed, coded, and recorded undergo quality control (exactitude and reliability), which is an essential condition to address the issue of validity. Once data quality has been verified, the data are subjected to the quantitative analysis plan (Table 4).

**Table 4:** Systematic observation process: QUAL-QUAN phase (II).

Systematic observation process: QUAL-QUAN phase (II)			
Dissertations Issues			
Review of marital life (Cuervo, 2014)	Teachers’ coordination meeting (Izquierdo, 1993/1995)	Interactive support group with women survivors (Roustan, 2010)	Lectures in university classrooms (Tronchoni, 2019)
Reliability			
Cohen’s Kappa Coefficient (*k*) (Cohen, 1960) Pearson correlation coefficient adapted to sequential data (*r*) (Holloway and Wampold, 1983)			
*k _intra_* = 1.0 *k _inter_* = 1.0	*r =* 0.99	*k _intra_* = 1.0 *k _inter_* = 0.70	*k _intra_* = 0.90
Descriptive analysis			
Binomial test: comparison of proportions	Tests χ^2^	Binomial test: comparison of proportions	Binomial test: comparison of proportions
Analysis of temporal regularities			
Lag	Lag	Lag	Lag
T-patterns	Polar coordinates	Polar coordinates	Polar coordinates

The quantitative analyses carried out, compatible with the observational design implemented (I-N/S/M), encompass the selected descriptive statistics in each study, together with lag sequential analysis, polar coordinate analysis and T-pattern detection (Anguera et al., 2021b).

It is important to bear in mind that the results of the analyses should answer the research questions, maintaining a coherent theoretical-methodological line that is rigorously argued and recognized by the scientific community of reference. The debate, which places value on the theoretical-methodological rationale of the usefulness of the considered studies, is complemented by the assessment of the participating people/groups in the observed conversational encounters with an evaluative-formative aim (see Figure 2).

*Computer programs.* The development of computer programs to code and record digital observational information and its subsequent quantitative analysis has made the task of systematic observation easier (Anguera et al., 2021b).

The manual introduction of the *ad hoc* observation instrument’s coding language and its application to the viewing of the video recordings carried out, also incorporates the automatic recording of the duration of the transactions previously delimited, identified and named by the observing analyst.

The obtained coding matrix comes from the diachronic-synchronous structure of the field formats, and the programs used for the quantitative analysis of the data matrix were, depending on the available access at the time of carrying out the research: Elag: Lagged event analyzer (Bakeman, 1985), SDIS-GSEQ (Bakeman and Quera, 1996), HOISAN 1.6.3.3.6 (Hernández-Mendo et al., 2012), Lince (Gabin et al., 2012), and Theme Coder v5 (Pattern Vision, 2002).

### 4.2 Simplified example of coding and recording rules

The logic of the decisions made to structure systematic observation provides the foundation upon which the QUAL-QUAN transformation (*quantitizing*) rests. The dynamic circularity (non-tautological) of this modality of method connect QUAL-QUAN-QUAL, enables researchers to obtain a holistic and integrated view of the problem under study by articulating quantitative and qualitative analyses through a single data matrix comprising categorical, operationalized, controlled, and diachronic data. Within this transformation process, it is particularly important to have a guide to orient the coding and recording process for the E/ME category list (see Table 5).

**Table 5:** Simplified example of coding-recording rules.

Scheme for the turn-taking dimension: speaker-turn category with or without listener overlap	Coding and recording rules (R) applied to the example	Izquierdo, 1993, p. 274	
Speaker A Speaker B Speaker C Speaker D Listener overlapping speech ()	R1*. The second-by-second temporal recording of speech does not necessarily correspond to the sequence of primary-speaker turns in the floor*. R2. *The simultaneity of voices at a given moment is not considered a simultaneous turn state if its duration is ≤ 2 s*. R3. *Listener overlap of ≤ 2 s. in turns ≥ 3 s. may serve two distinct functions: a request for the floor when the overlapping voice initiates the turn transition and takes the turn as the primary speaker. The second function concerns listener backchannels that signal ongoing monitoring of the current speaker’s turn*. R4. *Recording of the order (and duration) of speaker changes*. R5. *Recording of the order (and duration) of the succession of alternating and nested turns*.	Moment *u:v* 00:00 00:01 00:02 00:03 00:04 ------ ------ 00:17 00:18 ------ ------ 00:39 00:40	Vocal behavior - A A D D D D D (E) D D D E E
		*At second 17, D continues speaking while E overlaps. No multiple turn occurs* (*≤ 2 s*). There is no change in the primary or supporting speaker. The listener’s brief vocal behavior is coded as *auditory backchannels*. Sequential notation (Bakeman and Quera, 1996) of timed events (*u*) in the 40-s segment of dialogic interaction. A,1–2 D,3–16 D(E),17 D,18–28 E,39–40	

After identifying the constellation of the key decisions taken and the logic of the transformation applied, in the figure below we have summarized the content presented in sections 3 and 4 (see Figure 3).

**Figure 3:**
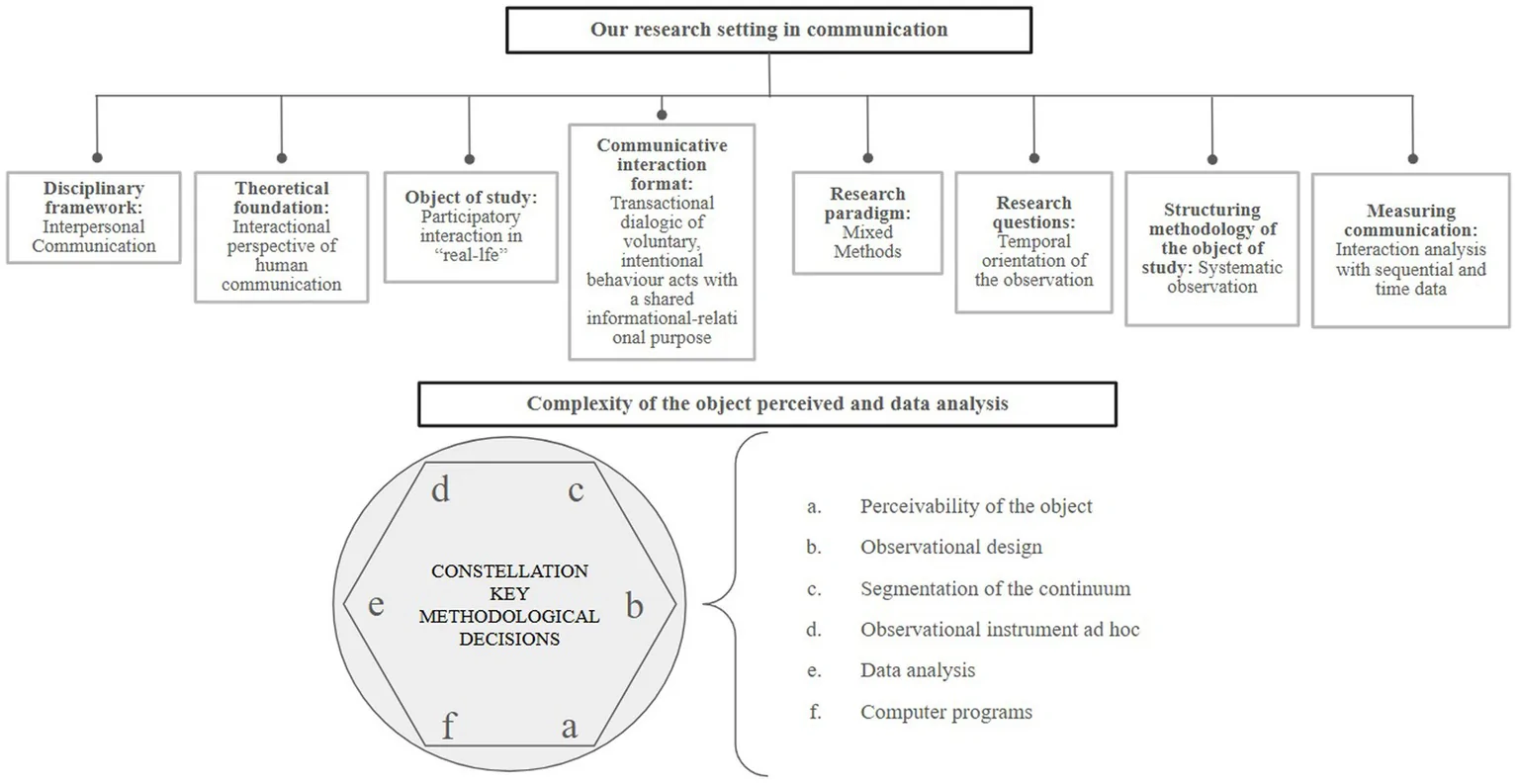
Organizing structures for the observation of the participatory interaction. Created by the authors.

## 5 Discussion

We frame the discussion to clarify that our preference for the mixed methods (MM) approach is not driven by the popularity of the third paradigm.

The call to systematize observational methods aims to advance the scientific procedures structuring the object-method relationship within the limits imposed by the nature of the study problem, the characteristics of the observational method used, and the available analytical techniques.

For this reason, we emphasize that the QUAL-QUAN-QUAL methodological procedure applied in observation studies of participatory interaction is uniquely suited precisely to the structural specifications of the object of study (Anguera, 2010).

The scientific quality required of both quantitative and qualitative studies of human communication aiming for publication in prestigious journals is evident when authors are asked to submit manuscripts that meet these generalizable requirements: “All research should provide descriptions of data collection and analysis procedures that are accurate, lucid, and comprehensive” (Slater et al., 2006, p. 373).

In highly systematized observational methodology, we have found the flexibility and rigor required to investigate human communication from an interactive and participatory perspective centered on the construction of meanings and relationships. The methodological advances incorporated from the final decades of the twentieth century to the present day have influenced every phase of the scientific observational methodology. Indeed, it is precisely the advances in the coding-recording process and in quantitative analysis based on primary parameters that have enabled the application of the connect integration pathway defined as “QUAN-QUAL” transformation through the matrix structuring of diachronic qualitative data (Anguera and Izquierdo, 2006). These data can be analyzed from three complementary perspectives: sequential lag analysis (Bakeman and Gottman, 1986), polar coordinate analysis (Sackett, 1980), and T-pattern detection (Magnusson, 2006, 2020).

It should be noted that effective qualitative data coding requires segmenting qualitative material into units and assigning them to a system of categories previously defined through theoretical criteria (a deductive approach), without overlooking the role of the inductive approach. As systematic observation methodologists emphasize: *“*Observer accuracy is a major concern, and typically considerable effort is spent training observers so that they will produce essentially similar records, given that they observed the same stream of behavior*”* (Bakeman and Gottman, 1987, p. 818).

In other words, the analytical observer is recognized as much for the intensity and directionality of their attention as for the demanding control exercised over the ideological load of their perceptive or cognitive representations of the subjects covered and the quality of observational research (Blanco-Villaseñor, 1993).

By placing the emphasis on the scientific role of the observer, the training and supervision of analysts observing human behavior within its contexts become particularly crucial. We wish to highlight both the importance of doctoral research committed to highly systematized observational methodology and the need for postdoctoral assistance from specialists in mixed methods systematic observation. The goal is to ensure that the “QUAL-QUAN-QUAL” structuring of observational methodology fulfills the requirements of the mixed methods approach: “Mixed methods studies […] require the highest level of methodological expertise to guide the conduct of the study throughout its different stages and ensure the application of the most appropriate procedures at each point.” (Anguera et al., 2018a, p. 2766).

The main contribution of the four doctoral dissertations considered in this research is that they offer recognized proof of having solved the research problem in accordance with the quality standards of systematic observation studies (Anguera et al., 2018b; Portell et al., 2015b) focused on the analysis of group interaction formed by dyads, small groups, or other dialogic groupings (Brauner et al., 2018).

This has been possible due to the deployment of instructive resources aimed both at understanding the nature of the third paradigm, and at using observational methodology with a full awareness of integrating qualitative and quantitative elements throughout the systematic observation process.

Given the points discussed, we may ask what innovation and contributions have emerged from the doctoral studies selected as mixed methods observational research.

Our work is not intended to provide innovation per se at the conceptual research level regarding the integration of qualitative and quantitative elements. Rather, the novelty lies in providing an organizing framework (see Section 4) for the controlled systematization of the practical observational process, not without its limitations, within the thematic domain of dialogic participatory interaction tied to everyday professional and non-professional tasks.

Sharing our reflective and reasoned experience alongside other convergent or related efforts in our thematic field under study, we think that may offer benefits that, of course, can only be fully realized through collaboration. This is how we have interpreted the call to action that opens this article: “It is time that methodologists catch up with practicing researchers! It is now time that all researchers and research methodologists formally recognize the third research paradigm and begin systematically writing about it and using it” (Johnson and Onwuegbuzie, 2004, p. 16).

In keeping with this open dialogue, all four dissertations have yielded journal publications, conference presentations, and seminars, alongside their direct impact on enhancing dialogic interpersonal competence.

To conclude, we take note of the advances in the use of supervised and unsupervised computational systems in the analysis of dialogic interaction. The opportunity to be able to apply the statistical technique of topic modeling with different options could be ideal in terms of indirect observation of the conversational task with multiple-case follow-up (Khalid and Wade, 2020). With advances in voice recording (voice source tracking and the use of lapel microphones) we can tackle the aim of segmenting a significant corpus of meetings into sequences of actions and phases, using audio information. We cannot ignore the advantages of the so-called *Smart Meeting Rooms* (Popescu-Belis and Bourlard, 2013) in that they provide an automated system for capturing, storing, structuring and indexing meetings; we believe they could become incorporated into certain social contexts (institutional and professional) as an ordinary conversational situation. A more widely expanding area could be that of the introduction of robots designed for social interaction. This new condition of a participating interlocutor widens the study of human-human interaction with the presence of a robot, while at the same time promoting the study of human-robot interaction (Rifinski et al., 2020).
